# Single-use flexible ureteroscopes: update and perspective in developing countries. A narrative review

**DOI:** 10.1590/S1677-5538.IBJU.2021.0475

**Published:** 2021-09-10

**Authors:** Eduardo Mazzucchi, Giovanni Scala Marchini, Fernanda Christina Gabrigna Berto, John Denstedt, Alexandre Danilovic, Fabio Carvalho Vicentini, Fabio Cesar Miranda Torricelli, Carlos Alfredo Battagello, Miguel Srougi, William Carlos Nahas

**Affiliations:** 1 Faculdade de Medicina da Universidade de São Paulo Hospital das Clínicas Seção de Endourologia São Paulo SP Brasil Seção de Endourologia, Divisão de Urologia, Hospital das Clínicas, Faculdade de Medicina da Universidade de São Paulo - FMUSP, São Paulo, SP, Brasil; 2 Western University Division of Urology London Canada Division of Urology, Western University, London, Ontario, Canada

**Keywords:** Meta-Analysis [Publication Type], Ureteroscopes, Kidney Calculi, Surgical Procedures, Operative

## Abstract

Flexible ureteroscopy is a well-established method for treatment of urinary stones but flexible ureteroscopes are expensive and fragile devices with a very limited lifetime. Since 2006 with the advent of digital flexible ureteroscopes a great evolution has occurred. The first single-use flexible ureteroscope was launched in 2011 and new models are coming to the market. The aim of this article is to review the characteristics of these devices, compare their results with the reusable devices and evaluate the cost-benefits of adopting single-use flexible ureteroscopes in developing countries.

**Materials and Methods:**

an extensive review of articles listed at PubMed and published between 2000 and 2021 was performed.

**Results:**

Single-use flexible ureteroscopes have a shaft with 65 to 68cm length and weight between 119 and 277g. Their deflection goes up to 300 degrees. Their stone-free rates vary between 60 and 95% which is comparable to reusable scopes and operative times ranges from 54 to 86 minutes which are lower when compared to reusable flexible scopes. Their costs vary between 800 and 3180 US dollars.

**Conclusion:**

single-use flexible ureteroscopes are lighter and have superior quality of image when compared to fiberoptic ones. There are no definite data showing a higher stone-free rate or less complications with the use of single-use flexible ureteroscopes. Each institution must perform a cost-benefit analysis before making the decision of adopting or not such devices depending on the local circumstances.

## INTRODUCTION

Flexible ureterorenoscopy is a well-established procedure for renal and ureteral stone management and reusable flexible ureteroscopes have been the standard device used for such procedures (1–3). A flexible ureteroscope (fURS) should be able to produce a good image, access the entire collecting system, have a good irrigation flow despite having a device in the working channel and be durable at a reasonable cost. These are simple requirements in theory, although they are not easy to achieve in daily practice, even with a variety of devices available in the market. Enormous progress in flexible ureteroscope technology has occurred in recent years, but problems with durability and costs persist. Considering these facts and aiming to mitigate these issues, manufacturers have launched single-use ureteroscopes. Currently, there are several single-use models in the market, and they have advantages and disadvantages over the reusable models.

Historically, the first ureteroscopy was described by Young in 1912 and, in 1964, the first ureteroscope was introduced by Marshall (4, 5). The clinical application of early devices was limited and allowed only diagnostic procedures as they lacked active deflection and a working channel. In 1987, Bagley introduced the flexible ureteroscope with a working channel, transforming ureteroscopy from a diagnostic to an interventional procedure (6). Another milestone in the flexible ureteroscope development was the introduction of digital technology in 2006 by Olympus - Gyrus - ACMI which greatly improved overall imaging quality (7). In the recent years, other technological advances, such as reductions in the scope’s caliber and improvement in the active deflection allowed for better surgical outcomes and a decrease in morbidity and surgical times (8). The most recent advancement in ureteroscope technology was the introduction, in 2011, of the first single-use ureteroscope (Polyscope^TM^) by Lumenis which utilized a reusable fiberoptic bundle that could be attached to disposable flexible catheters (9). The model was not widely adopted due to its low efficacy especially for lower pole stones owing to limited deflection capabilities reaching a 69% success rate in such cases (10). In January 2016, Boston Scientific introduced the first digital single-use ureteroscope, the LithoVue™ (11). This opened a new era for the development of new single-use devices transforming flexible ureteroscopy and retrograde intra renal surgery (RIRS).

The aim of this article is to review the current literature on single-use flexible ureteroscopes, including advantages and disadvantages over reusable ureteroscopes and analyze their cost-benefit in the context of developing countries.

## MATERIALS AND METHODS

**Figure 1:**
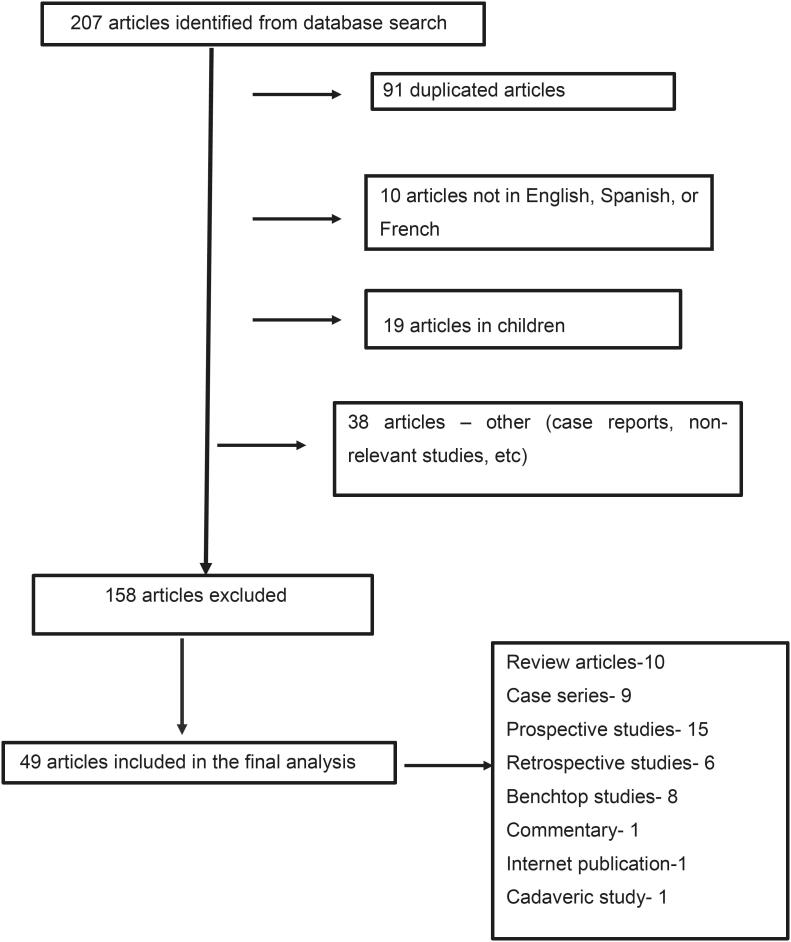
Flow diagram of evidence acquisition in a review on single-use and reusable flexible ureteroscopy.

A PubMed database search was conducted in January-February 2021 using the following Medical Subject Heading (MeSH) terms in several combinations: single-use flexible ureteroscope, disposable flexible ureteroscope, cost of flexible ureteroscopy, cost of single-use ureteroscope, durability of reusable flexible ureteroscopes, ureteroscopy, ureteropyeloscopy, ureterorenoscopy. We included original articles published between January 2000 and January 2021, in English, French or Spanish languages. Additionally, web pages from manufacturers were included. Studies involving children and case reports were not included. Initially, 208 articles were reviewed, 158 studies were excluded due to reasons shown in the flowchart. Therefore, in our final analysis, 51 articles were included. The flowchart is shown in Figure-1.

### Potential advantages of single-use ureteroscopes

Single-use flexible ureteroscopes have advantages and disadvantages when compared to reusable ureteroscopes. Reusable ureteroscopes are expensive devices requiring a high initial investment which includes the purchase of light source, camera, image processor, monitors, and cables, among others. Also, processing the instruments after each use requires specialized personnel and facilities and involves cleaning, decontamination, drying, testing, sterilizing, and packing. This procedure is time and financially consuming. According to Isaacson et al., the processing of a ureteroscope after surgery takes an average of 229 minutes while the mean case duration of flexible ureteroscopy for treating a renal stone is, on average, 64 minutes at the same hospital (12). Processing a single flexible ureteroscope costs 96 US dollars, with the cost of a single Sterrad^TM^ sterilization cassette being responsible by 25% of the total cost (12). Of course, these costs were calculated in a single North American Institution and may vary in the different countries around the World. Another issue is the breakage of flexible scopes either during surgeries or processing after surgery. It is common sense that instruments manipulated by many surgeons and/or by inexperienced urologists have a higher chance of damage, especially if not properly supervised (13). Mishandling during reprocessing and out of the operating room can be responsible for 7.7% to 22% of damage to flexible ureteroscopes (14, 15). Repairing flexible ureteroscopes is difficult, and at times almost impossible in developing countries. The durability of a flexible ureteroscope varies significantly according to multiple factors such as the complexity of the cases treated, the number of surgeons that manipulate the scope, the sterilization method, and the presence of specialized personnel for handling the instrumentation. When handled by a single surgeon, a reusable scope can reach up to 159 procedures (16). According to the literature, a fiberoptic scope needed repair after a mean of 21 cases, and a digital scope after 10 to 21 cases with a mean cost of 848 US dollars per repair (17–19) with eleven days being the mean time for repairing the scopes. The time for repairs can vary widely depending on the region/country involved (17–19). The durability of refurbished flexible ureteroscopes though, is inferior when compared to a new device with a mean life of only seven procedures (20). None of these issues are relevant for single-use ureteroscopes and surgeons have the additional advantage of always using a brand-new device.

Another discussion point is regarding infection. The occurrence of acute pyelonephritis following ureteroscopy is 2.4%, which is low, but not negligible (21). A study published in 2017 by Ofstead et al., showed the presence of bacteria, hemoglobin and protein inside reusable ureteroscopes after manual cleaning and sterilization by hydrogen peroxide gas but there are no clinical data proving the influence of these findings in the occurrence of post-operative infections following ureteroscopy (22). Despite these data, single-use ureteroscopes did not decrease the occurrence of infectious complications after ureteroscopy according to a recently published study showing that the scope is probably not the main source of post-operative infections in ureteroscopy cases (23).

Another point refers to a potential higher success and stone-free rate with single-use scopes, especially when dealing with difficult cases such as acute-angle lower pole stones and abnormal kidneys such as horseshoe and pelvic kidneys. The literature is still scarce and fails to show any significant difference between reusable versus disposable instruments (24). Many studies have examined that kidneys with a steep lower pole angle represent a risk factor for ureteroscope damage and unfavorable results encouraging the use of single-use flexible ureteroscopes in such cases (25, 26). This can be especially true when working with an extensively used scope where deflection and vision are already impaired, which is common in developing countries.

Operative time should also be considered when discussing the choice for a flexible ureteroscope, since longer operative times impact directly in costs. Both reusable and disposable digital flexible ureteroscopes present a 20% shorter operative time when compared to fiberoptic scopes (13). In a series published by Somani et al., the cases performed with the Olympus URF-V™ had an operative time nine minutes shorter than the cases where an Olympus URF-P5™ was used (27). Similar results were observed by Usawachintachit et al. comparing the LithoVue™ with reusable flexible ureteroscopes in stone cases (57.3±5.1 vs. 70.3±36.9 minutes, p <0.005) (28).

### Single-use flexible ureteroscopes: technical characteristics

**Figure 2:**
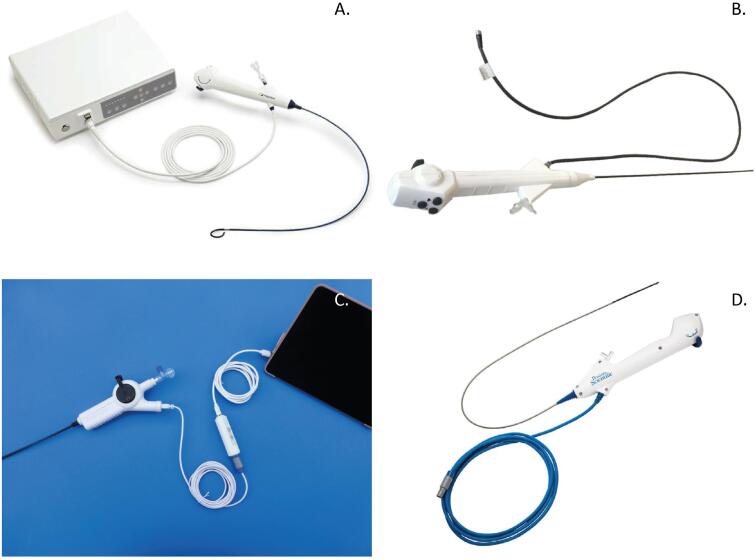
shows the different types of single-use flexible ureteroscopes.

Generally, single-use flexible ureteroscopes have similar physical characteristics (Figure-2). The shaft length varies between 64.5 and 68cm; the shaft size ranges 9.0 to 9.5Fr with the tip diameter between 7.4 and 9.5Fr. The working channel is commonly 3.6Fr. The illumination is by LED (light-emitting diode) and the camera sensor type, which is an electronic chip that converts photons to electrons for digital processing, is CMOS (complementary metal oxide semiconductor) in the majority of devices. Deflection is dual, reaching 280 degrees up and downward. They are lighter when compared to reusable scopes: LithoVue™ weighs 277g against 344g for the Storz Flex X2™ and 942g for the Olympus URFV 2™ (29). One of the new models, the Neoflex™ (Neoscope^TM^) has an advantage regarding portability. The scope can be connected by an attached USB 2.0 cable directly to any high definition (HD)-compatible video monitor or personal computer. This way, the ureteroscope does not require a separate processor or light source. This connectivity feature enables Neoflex^TM^ to be completely portable compared with other single-use flexible ureteroscopes that require an endoscopic video tower to function. Its portability is a major advance as this ureteroscope can be used in diverse environments, including remote and developing areas of the World (30) (Figure-2). The Axis™ single-use ureteroscope showed a 300-degree deflection in an in vitro study performed by Whelan et al. and this deflection was reduced by only 2% after 200 deflections, proving its high level of resistance which can be useful in demanding cases (31).

Data on technical characteristics of several single-use flexible ureteroscopes are summarized in Table-1.

**Table 1:** Shows the comparison of the physical (weight, shaft length, working channel) and functional(deflection, irrigation flow) characteristics of the single-use flexible ureteroscopes currently disponible in the market (modified from Scotland et al. (7), Proietti et al. (28), Whelan et al. (30) and Dragos et al. (31).

	LithoVue™	Uscope UE3022™	Neoflex™	Dornier - Axis™	WiScope™
Shaft length (cm)	68	65	68	66	67
Tip outer diameter (Fr)	9.5	9.0	9.0	8.5	7.4
Deflection up/down (degree)	270	270	280	275	275
Working channel (Fr)	3.6	3.6	3.6	3.6	3.6
Irrigation flow empty channel (mL/min)	42	52	40	NA	49
Illumination	LED	Fiberoptic fiber	LED	LED	LED
Imager technology	CMOS	CMOS	CMOS	CMOS	CMOS
Weight (g)	277	147	119	160	200
Connector type	Round and 8 pins	Flat	USB	USB	Round

### In vitro and clinical comparisons between reusable and single-use flexible ureteroscopes

Somani et al. compared the clinical results between fiberoptic and digital reusable flexible ureteroscopes for the treatment of renal stones. The stone-free rates were 86% and 88% (p-value not significant) for fiberoptic and digital ureteroscopes, respectively and the complication rates were 1% and 0.9% for both ureteroscopes. The operative time was significantly shorter for digital flexible ureteroscopes (44 min vs. 54 min for fiberoptic scopes, p <0.05) (27). More recently some studies comparing the various characteristics of reusable and single-use scopes in vitro and in vivo have been published.

Irrigation is of utmost importance in flexible ureteroscopy to facilitate adequate vision of the operating field. Two studies compared irrigation parameters between reusable and single-use scopes. In the first, Marchini et al. compared the LithoVue™ and Pusen™ with the Storz Flex X2™, a fiberoptic reusable flexible ureteroscope immensely popular in developing countries. The irrigation flow was superior in the single-use scopes when compared to the reusable scopes with the working channel empty or when instruments such as a 200µm laser fiber or a 1.3Fr basket were inserted (32). In a similar study, the Neoscope^TM^ showed better irrigation in all situations (empty channel, 200µm laser fiber and 1.9Fr basket inserted) when compared with digital reusable Storz Flex XC™ and fiberoptic Wolf Cobra™ (32). These data were confirmed by another study conducted by Dragos et al. who compared the irrigation flow between four single-use and four reusable flexible ureteroscopes. The only exception was the Wolf Cobra which has two working channels (one 3.6Fr and another 2.4Fr) and it is not affected by the insertion of instruments (33).

Imaging quality was evaluated by Talso et al. who conducted an in vitro study comparing the fiberoptic flexible ureteroscopes (Olympus P6™, Storz Flex X2™) and digital (Olympus URF-V™ and URFV2™, Storz Flex XC™, Wolf Cobra vision™) reusable devices with the LithoVue™. Two of the reusable digital scopes (Storz Flex XC™ and Olympus URFV™) provided better images than the LithoVue™. The LithoVue™ imaging quality though, was superior to Olympus URFV2™ and Wolf Cobra vision™. All of them were significantly better than the fiberoptic flexible scopes in the different settings of the evaluation (34). In another in vitro study comparing LithoVue™ with the Storz Flex XC™ and the Wolf Cobra fiberoptic scope™, the authors concluded that the image resolution was similar in the Flex XC™ and in the LithoVue™ and it was 40% better than in the Cobra reusable scope™ (34). In a third study, conducted by Dragos et al., four single-use flexible scopes (LithoVue™- Boston Scientific, Uscope^TM^- Pusen, Neoscope^TM^- Neoflex™ and Shao GangTM- You Care) were compared to four reusable flexible ureteroscopes (Flex XC^TM^- Storz, URFV2^TM^- Olympus, Cobra™ and Boa vision^TM^- Richard Wolf). The authors concluded that the field of view was slightly better in the LithoVue™ but the depth of view, resolution and color reproducibility were better for the reusable scopes tested (33). In conclusion, it seems clear that all digital scopes (single-use or reusable) provide better imaging quality when compared to fiberoptic scopes. In the same study by Dragos et al., deflection was compared among the eight scopes. A 200μm laser fiber had the least impact on deflection (2.198 degrees) for the single-use flexible ureteroscope, and the 1.5Fr retrieval basket (1.971 degrees) for the reusable scopes. The PTFE coated guidewire determined the highest impairment on deflection for all flexible ureteroscopes (31). In almost all settings, the single-use scopes had better deflection than their reusable counterparts, but reusable flexible ureteroscopes achieved superior deflection compared to the single-use scopes when larger caliber instruments were inserted through the working channel (365μm laser fiber or guide wires - both PTFE and nitinol). After the tests, almost all of the single-use flexible ureteroscopes had some deflection loss but none of the reusable scopes presented with deflection impairment (33).

Some of these in vitro findings were confirmed in a fresh-cadaver study performed by Proietti et al. In this study, LithoVue™ was compared with the Olympus P5^TM^ fiberoptic scope and the URFV™ digital scope in four renal units of fresh female cadavers regarding accessibility to the kidney and navigation of the entire collecting system with and without ureteral access sheath (UAS). Access to the lower pole was measured evaluating the deflection of the ureteroscope with an empty working channel and with the presence of different baskets and laser fibers. LithoVue™ performed similar to the two reusable devices regarding maneuverability, navigation of the entire collecting system, and angle of deflection in the lower pole with or without devices inside the working channel (35).

Usawachintachit et al., prospectively compared the stone-free rate (in this case the complete absence of residual fragments), the occurrence of insignificant residual fragments (≤2mm), and the presence of significant fragments (>2mm) between the fiberoptic device Olympus P6^TM^ and the LithoVue^TM^. The results were 60.0%, 12.5%, 27.5% for LithoVue^TM^, and 44.7%, 13.2%, 42.1% for URF-P6^TM^ (p=0.36), with a tendency towards better outcomes with the single-use scope. The complication rate was lower in the LithoVue™ group (5.4%) compared to 18% in the URF-P6^TM^ group (p <0.05) (28).

Mager et al., in another clinical study, evaluated two groups of 68 patients. In the first group, surgery was performed using reusable flexible ureteroscopes from Storz (models Flex X2S^TM^ and Flex XC^TM^) while the second group was treated using the LithoVue™. The stone-free rates were 82% and 85% for reusable and single-use scopes, respectively. There were no differences in operative time and fluoroscopy time. Patients treated using the single-use device LithoVue™ though, had a higher complication rate compared to those operated with the reusable scopes (17% vs. 7%, p=0.06) (36).

Salvadó et al. reported the results of 71 procedures for upper ureteral and renal stones with a mean size of 11.4mm using the Uscope - Pusen 3022^TM^ (37). The mean operative time was 57min and the stone-free rates were 98% for stones smaller than 10mm, 95% for stones 10-20mm and 78% for stones larger than 20mm. The complication rate was 9% and complications were all minor according to the Clavien-Dindo classification (38, 39). Average fluoroscopy time was 74 seconds. These numbers are comparable to those published by the Clinical Research Office of the Endourological Society (CROES) study in the predisposable era (39). The same author compared the stone-free rates of the Pusen 3022^TM^ with the Wolff Cobra^TM^ reusable scope for treatment of lower pole stones and found no significant differences (95% for the Pusen^TM^ and 88.2% for the Cobra^TM^, p=0.1). The operative and the fluoroscopy times were both significantly shorter for the single-use ureteroscope (56.1±34.8 and 77±37.4 minutes, p=0.01 and 66.1±60.9 and 83.4±44.9 seconds, p=0.02 for the Pusen^TM^ and Cobra^TM^, respectively). There were no surgical complications reported in this study (40).

In a more recent study, Kam et al. conducted a prospective and randomized comparison among the LithoVue™, the Pusen 3022^TM^, and the Olympus URFV2^TM^ reusable digital scopes in 150 patients. Scope failure occurred in 14 of 150 procedures (9%) and was similar among scopes: three failures with the LithoVue™ (5%), six failures for the Pusen^TM^ (10%) and five for the Olympus URFV2^TM^ (8%) (41). Visibility and maneuverability were better for the Olympus URFV2™ when compared to both single-use flexible ureteroscopes. Despite these technical differences there were no differences regarding operative time, complications and necessity for a second-look procedure demonstrating that all scopes performed satisfactorily in the clinical setting (40). Results of flexible ureteroscopy for treatment of renal stones with single-use and reusable flexible ureteroscopes are summarized in Table-2.

**Table 2:** shows the comparison of results (stone-free rates, complications and operative time) of renal stone treatment with single-use and reusable flexible ureteroscopes.

	Stone-free rates (%)			
	Single-use	Reusable		p
Usawachintachit et al., 2017 (28)	60	44.7		0.36
Mager et al., 2018 (36)	82	85		0.8
Salvadó et al., 2019 (40)	95	88.2		0.1
Kam et al., 2019* (41)	87	90		ns
	**Complication rates (%)**			
Usawachintachit et al., 2017 (28)	5.4		18	< 0.05
Mager et al., 2018 (36)	17		7	0.06
Kam et al., 2019 (41)	29		19	ns
	**Operative times (min)**			
Usawachintachit et al., 2017 (28)	54.1 ± 25.7		64.5 ± 37.0	< 0.05
Mager et al.,2018 (36)	76.2		76.8	0.9
Salvadó et al., 2019 (40)	56.1		77	<0.01
Kam et al., 2019 (41)	86.1		72.3	ns

* Stone-free rate calculated based on the need for a second look pyeloscopy.

The treatment of urothelial tumors should also be taken into consideration when comparing advantages and disadvantages of single-use flexible ureteroscopes. According to what we have shown above, digital ureteroscopes achieve better quality of image when compared to fiberoptic scopes and this is a fact of significant importance for the endoscopic treatment of such tumors (42, 43). Reusable digital scopes have image enhancement technologies like the NBI (Narrow Band Image) from Olympus and Image 1-S^TM^ technology from Karl Storz. NBI is basically a color filtering of the light emitted by the ureteroscope which enhances the visibility of highly vascularized tissues. Compared to white-light ureteroscopy, real-time NBI technology increases tumor detection rate by 22% (44, 45). NBI is a trademark from Olympus and requires an NBI-able light source and a corresponding NBI-able ureteroscope capable of digital reprocessing. NBI is currently solely integrated to the Olympus URF-V™, URF-V1™ and URF-V2™.

The Image 1-S™ technology (formerly SPIES) involves re-processing of the image captured by the digital image sensor and, on the contrary of NBI, does not rely on a modified light source spectrum. Image 1-S™ technology offers enhanced contrasting of digitalized images providing better imaging quality. With this technology any light source can be used. The Image 1-S™ technology allows five re-processing modes, of which the “Clara+Chroma” mode has been shown to reach the highest quality of image. Whether this improvement impacts on tumor detection rate during ureteroscopy has not been evaluated in any study to date. The Image 1-S™ technology is currently solely integrated to the Storz Flex XC™ but theoretically, may be applied to any fiberoptic ureteroscope when an Image 1-S™ camera is appended at the instrument’s eyepiece (45, 46).

In conclusion, for stone treatment, the data currently available in the literature demonstrate that single-use flexible ureteroscopes have similar performance to the reusable scopes in the majority of the studied parameters with some advantage in terms of quality of image for the digital reusable scopes and advantages regarding irrigation flow and deflection for the single-use instruments. To date there are no definite clinical data proving advantages of one or another in terms of better clinical results (higher stone-free rates, lower complication rate, less fluoroscopy time, less related infection) but the idea of always using a new device during surgery and having less concerns regarding the quality of image, deflection, and breakage during or after procedures seems very attractive. On the other hand, according to the current literature one can conclude that reusable digital flexible ureteroscopes present some advantages in the treatment of urothelial tumors thanks to technologies that enhance visibility and increase tumor detection, but this has not yet been proven.

### Cost analysis of single-use ureteroscopes

As discussed previously, there are no significant differences among single-use and reusable digital flexible ureteroscopes regarding the stone-free rates, but the operative time is significantly reduced according to the current literature as showed above. Nonetheless, there are significant differences in terms of costs according to the device’s mode of usage across different countries.

Flexible ureteroscopes are recognized as fragile and expensive devices. It is important to keep in mind that beyond the cost of the ureteroscope per se, there are other expenses for building an endourological operating room like the light source, camera, image processor, monitors, and cables, among others. Although these apparatuses are much more durable, they require a high initial investment from hospitals which can reach prohibitive values especially in lower income countries.

On the other hand, the cost of the single-use flexible ureteroscope comprises only the cost of the scope once the processor/image unit is provided by the manufacturer or its representative. For reusable scopes, a simplified equation was created to estimate its cost:

Cost of a reusable scope=(original purchasing cost of reusable fURS) + [(repair cost per case/average number of cases before failure) (x)] + [(reprocessing cost per case) (x)] + [(cost of labor per case) (x)], where x=the number of cases (17).

It must be acknowledged that in most developing countries there is limited availability and capacity for repair of reusable flexible ureteroscopes. The final cost will be the initial cost for purchase divided by the number of cases per scope plus the total costs of reprocessing the instrument during its lifetime. As a result, surgeons and paramedical staff must be extremely committed to the correct use and processing of scopes otherwise, many hospitals will not be able to afford these procedures. Complete reprocessing of a scope after a procedure involves cleaning and decontaminating the instrument itself and its storage case with appropriate detergents, drying, performing a leak test, and sterilizing in the Sterrad™ before sending it to storage or to another procedure as mentioned earlier in this article. In Germany, a study analyzed the costs of 423 diagnostic and therapeutic ureteroscopies during a four-year period comparing reusable scopes (Storz Flex - X2^TM^ and Olympus URFV^TM^) with the LithoVue™. Each procedure performed with reusable scopes cost 503 euros while those performed with single-use devices resulted in 1000 euros expense (47). Conversely, an US study compared the Olympus P6^TM^ with the LithoVue^TM^ in a one-week trial that included all costs of reprocessing the scopes. The authors reported a cost of 2.799 US dollars for each procedure performed with the Olympus P6^TM^ and 2.852 US dollars for those performed with the LithoVue™ (48). In a third study, also from the United States, the authors evaluated the costs of 160 procedures performed with the Storz Flex XC^TM^ and compared with the potential costs of surgeries performed with the LithoVue™. The cost of each procedure with the Flex XC^TM^, excluding the costs of purchasing, was 848 US dollars. The authors concluded that, in their center, single-use scopes were cost-effective only if less than 99 procedures were performed each year and recommended single-use devices for low-volume centers (17).

The acquisition cost of each ureteroscope varies throughout the World. Moreover, high volume hospitals can negotiate better capital purchase pricing which can include repairing or substitution of damaged scopes at no cost or lower prices. The same rationale can be applied to single-use devices where manufacturers or representatives charge lower prices for a higher volume of devices. Temporary variations of costs are also observed according to the model of the scope. Single-use flexible ureteroscopes costs range from 800 to 3.180 US dollars and reusable scopes from 13.000 to 85.000 US dollars (49).

Based on the above, one can conclude that the decision of adopting the use of single-use or reusable scopes, or a hybrid model will depend on the conditions of purchase between the hospital and the manufacturer or its representative and the volume of cases in each institution. Basically, there are three models of cost-analysis for adopting single-use scopes. The first is in high-volume centers where the high number of scopes used leads to a more attractive arrangement with the supplier and a reduction in the scopes processing time resulting in a final gain to the institution. The second model is for hospitals with a very low volume of surgeries where the initial investment and maintenance costs of the facilities where a flexible ureteroscopy could be performed is not cost-effective. The third model is the adoption of a hybrid system where single-use scopes are used in those cases where the chance of breakage of a reusable scope is higher (examples: a steep angle between the ureter and the inferior calyx, anomalous kidneys like horseshoe or pelvic kidneys, stones larger than 2cm) (17, 26, 48, 49). Again, the adoption of each model will depend on each country, its health care system, and institutional models.

In conclusion, the current established concepts are that single-use flexible ureteroscopes are lighter, have excellent deflection and irrigation parameters. They have superior quality of image when compared to fiberoptic scopes but are inferior to reusable digital instruments. Additionally, single-use flexible ureteroscopes do not have enhancement image technologies present in reusable digital scopes that can make difference in the treatment of urothelial tumors. There are no definite data regarding a higher stone-free rate or less complications with the use of single-use flexible ureteroscopes, but the operative times are shorter when compared to reusable ureteroscopes. Furthermore, they can be of great value in difficult cases where the chance of instrument damage is higher, especially in lower pole stones. Regarding costs, each institution must perform a cost-benefit analysis before making the decision of adopting or not such devices depending on the local circumstances.
